# Association of Early Serum Phosphate Levels and Mortality in Patients with Sepsis

**DOI:** 10.5811/westjem.58959

**Published:** 2023-04-28

**Authors:** Lauren Page Black, Michael Mohseni, Ehsan Shirazi, Kasondra Hartman, Carmen Smotherman, Charlotte Hopson, Elizabeth DeVos, Rosemarie Fernandez, Johnathan Sheele, Faheem Guirgis

**Affiliations:** * University of Florida College of Medicine - Jacksonville, Department of Emergency Medicine, Jacksonville, Florida; † Mayo Clinic, Department of Emergency Medicine, Jacksonville, Florida; ‡ University of Florida Health Jacksonville, Center for Data Solutions, Jacksonville, Florida; § University of Florida College of Medicine, Department of Emergency Medicine, Gainesville, Florida

## Abstract

**Background:**

Metabolic derangements in sepsis influence phosphate levels, which may predict mortality outcomes. We investigated the association between initial phosphate levels and 28-day mortality in patients with sepsis.

**Methods:**

We conducted a retrospective analysis of patients with sepsis. Initial (first 24 hours) phosphate levels were divided into phosphate quartile groups for comparisons. We used repeated-measures mixed-models to assess differences in 28-day mortality across the phosphate groups, adjusting for other predictors identified by the Least Absolute Shrinkage and Selection Operator variable selection technique.

**Results:**

A total of 1,855 patients were included with 13% overall 28-day mortality (n=237). The highest phosphate quartile (>4.0 milligrams per deciliter [mg/dL]) had a higher mortality rate (28%) than the three lower quartiles (P<0.001). After adjustment (age, organ failure, vasopressor administration, liver disease), the highest initial phosphate was associated with increased odds of 28-day mortality. Patients in the highest phosphate quartile had 2.4 times higher odds of death than the lowest (≤2.6 mg/dL) quartile (P<0.01), 2.6 times higher than the second (2.6–3.2 mg/dL) quartile (P<0.01), and 2.0 times higher than the third (3.2–4.0 mg/dL) quartile (P=0.04).

**Conclusion:**

Septic patients with the highest phosphate levels had increased odds of mortality. Hyperphosphatemia may be an early indicator of disease severity and risk of adverse outcomes from sepsis.

## INTRODUCTION

Patients with sepsis have life-threatening organ dysfunction caused by a dysregulated host response to infection.^1^ Septic shock is the most severe manifestation of sepsis syndrome with increased mortality due to circulatory and metabolic abnormalities.^1^ Approximately 1.7 million adults are hospitalized with sepsis per year in the United States, with a mortality rate of 15.6%.^2^ Early recognition and diagnosis of sepsis remain challenging due to the nonspecific diagnostic criteria for the syndrome. Consensus guidelines recommend prompt resuscitation with intravenous crystalloid, early administration of broad-spectrum antibiotics, and vasopressors to support blood pressure in the setting of septic shock.^3^ Elevated serum lactate levels are indicative of cellular dysfunction in sepsis, making hyperlactatemia a useful marker of sepsis severity.^1^,^4^–^6^

Other metabolic derangements occur in critically ill sepsis patients, including changes in phosphate levels. Phosphorus is a component of cell membranes, nucleic acids, and nuclear proteins. It is required for energy metabolism and intracellular signaling, and it plays a key role in regulatory mechanisms, generation of adenosine triphosphate, acid-base homeostasis, and oxygen release by hemoglobin.^8^,^9^ Phosphate derangements are, therefore, commonly expected in critically ill patients.

Hypophosphatemia has been described in sepsis, metabolic or respiratory alkalosis, refeeding syndrome, and ketoacidosis, as well as after major trauma or surgery.^10^ It has also been associated with increased morbidity and mortality in critically ill patients.^11^–^13^ Several studies suggest hypophosphatemia may be an early indicator of infection and increased mortality in sepsis.^14^,^15^ However, more recent studies found associations between hyperphosphatemia and increased mortality in critically ill patients with sepsis and septic shock.^16^,^17^ The direction of early phosphate derangement (high or low) and its predictive ability for mortality in sepsis remains unclear. Our primary objective in this study was to determine the association of initial phosphate levels (measured in the first 24 hours) in septic patients with 28-day mortality.

## METHODS

### Data Source

We conducted a retrospective analysis using an existing dataset of adult patients admitted for sepsis at University of Florida Health Jacksonville, an urban, not-for-profit academic medical center. The parent study evaluated hospital sepsis outcomes before and after the implementation of a quality improvement program.^18^ The parent study was a retrospective analysis of patients identified by diagnosis codes for sepsis in the University of Florida Health Jacksonville electronic health record system.^18^ Trained research coordinators worked in concert with the biostatistician and clinician team to clean and curate the data for analysis. Our approach and reporting followed STROBE guidelines.^19^ The study was approved by the University of Florida Institutional Review Board with a waiver of informed consent.

The original dataset included patients admitted with any one of 28 explicit *International Classification of Diseases, 9^th^ Revision, Clinical Modification* codes for sepsis (see Supplemental Information) and two or more systemic inflammatory response syndrome (SIRS) criteria between October 2013–May 2016. In 2016, the Third International Consensus Definitions for Sepsis and Septic Shock (Sepsis-3) deemphasized SIRS criteria, defining sepsis as a dysregulated response to infection. 1,4 Sepsis-3 operationalized this consensus definition as an increase in the Sequential Organ Failure Assessment (SOFA) score of two or more points.^4^ Therefore, we a priori planned a sub-analysis limited to patients with a SOFA score of two or more.

**Population Health Research Capsule**

What do we already know about this issue?

*Phosphate derangements are common in critically ill patients and may be an early indicator of infection and increased mortality in patients with sepsis*.

What was the research question?

*Our objective was to determine the association of initial phosphate levels in septic patients with 28-day mortality*.

What was the major finding of the study?

*Patients with the highest phosphate levels (4.0 mg/dL) had a higher mortality rate than those in the three lower quartiles (P<0.001)*.

How does this improve population health?

*Hyperphosphatemia may be an early indicator of disease severity and risk of adverse outcomes from sepsis*.

### Exclusion and Inclusion Criteria

**Figure 1:**
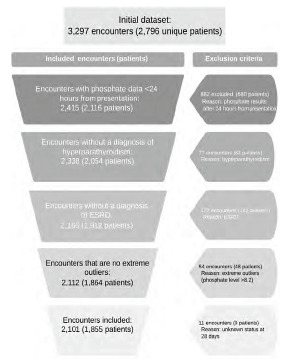
Patient inclusion flow diagram. *ESRD*, end-stage renal disease.

Patients were included in this study if they met the above study criteria for sepsis and they were in the parent dataset and had a phosphate lab result within the first 24 hours of emergency department (ED) presentation. We excluded patients who had a diagnosis of hyperparathyroidism, hypoparathyroidism, or end-stage renal disease (ESRD), as well as those patients who did not have a phosphate lab result within 24 hours of ED presentation (Figure 1).

### Data Collection

We collected demographic information, clinical data, vital signs, SOFA scores, and lab values for all patient encounters. Phosphate levels collected within 24 hours of ED presentation were included for analysis. Only the initial phosphate laboratory result from each encounter was included in the analysis, as subsequent values may have been influenced by phosphate repletion, and phosphate repletion data was not available. We excluded phosphate results (reported in milligrams per deciliter [mg/dL]) that were extreme outliers (greater than 3 interquartile range [IQR] above the third quartile) among all phosphate lab results from the study.^20^ There were no extremely low outliers. The quartiles of the initial phosphate values were used as the boundaries of the four phosphate quartile groups. The primary outcome was 28-day mortality.

### Statistical Analysis

We summarized data using medians (first quartile, third quartile) for continuous data and using counts and percentages for categorical data. Chi-square and Wilcoxon rank-sum tests were used to compare the baseline characteristics among the phosphate quartile groups. For univariate analyses, we used data from the first encounter if the patient had multiple encounters during the study period. Repeated-measures mixed-models were used to identify predictors of 28-day mortality. We used data from all encounters for the 28-day mortality multivariable regression model with repeated-measures mixed-models to account for repeated encounters. Candidate predictors in the model were phosphate quartile group; age at first encounter (years); gender (biologic sex); race (Black, White, other); vasopressor use (yes/no); mechanical ventilation (yes/no); history of diabetes (yes/no); history of liver disease (yes/no); initial creatinine level during the encounter; initial calcium level during the encounter; total SOFA scor; and the interaction between creatinine and SOFA score.

Missingness was under 1% for all variables included in the logistic regression model. As a prescreening step to discard the least important model terms, the least absolute shrinkage and selection perator (LASSO)^21^ and least angle regression^22^ methods implemented in the GLMSELECT^23^ SAS procedure were used. The LASSO method is a penalized regression method that considers all candidate variables and reduces the coefficients of non-important variables to zero, thereby removing them from the final model. It allows us to perform comprehensive variable selection from the candidate variables and reduce multicollinearity. We determined the optimal covariance structure by fitting several covariance structures and determining the one with the lowest corrected Akaike information criterion.^24^ We estimated differences between groups using adjusted odds ratios (aOR), along with 95% confidence intervals. We used area under the receiver operating characteristic (ROC) curve to assess the performance of the predictive model. The level of significance was set at 5%. We performed all analyses using SAS version 9.4 for Windows (SAS Institute, Inc; Cary, NC).

## RESULTS

We reviewed data from 3,297 encounters in the original dataset, representing 2,796 unique patients, for inclusion in this study. The primary reason for exclusion was a lack of phosphate levels within the first 24 hours of ED presentation (882 encounters). Other reasons for exclusion included a history of ESRD or parathyroid disorders. Finally, we excluded extremely high outlier phosphate values (phosphate >8.2 mg/dL). The final study population included 2,101 encounters, representing 1,855 unique patients (Figure 1).

The 1,855 patients admitted for sepsis had a median age of 60 years (Table 1); 51% of patients were female, 48% were Black, 47% were White, and 5% were other races. The median initial SOFA score was 2 (0, 5). Table 1 displays demographics, comorbidities, and disease severity for enrolled patients. The overall 28-day mortality rate was 13% (237).

Among all encounters for sepsis, the median phosphate level was 3.2 mg/dL, the bounds of first and third quartiles were 2.6 and 4.0 mg/dL, respectively (Table 2). The median time to initial phosphate level was 2.87 hours (quartiles 0.7, 12.8). Table 2 presents the number of patients, the mortality rate, and the distributions of phosphate levels within each quartile group by unique patients. Mortality differed among phosphate quartile groups (*P* <0.001). The greatest mortality rate (28%, 113) occurred in the quartile with the highest phosphate levels (> 4.0 mg/dL). The quartile with the lowest phosphate levels (≤ 2.6 mg/dL) had the lowest mortality rate (7%, 38). The second and third quartiles had mortality rates of 9% (39) and 10% (47), respectively.

As shown in Table 1, both comorbidities and disease severity varied among phosphate quartile groups. Compared to other quartile groups, patients in the highest quartile were older and more likely to have a history of congestive heart failure or myocardial infarction. There was also a significant difference in rates of chronic obstructive pulmonary disease across phosphate quartiles. Initial creatinine and lactate levels were significantly higher among patients with higher phosphate levels. Initial total SOFA scores increased with phosphate quartiles, with the highest quartile experiencing the most pronounced degree of organ failure. Patients in the highest quartile were also more likely to require mechanical ventilation and vasopressor support.

### Multivariable Analyses

**Figure 2:**
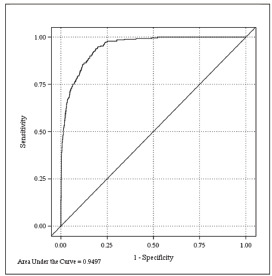
Receiver Operating Characteristic (ROC) curve for 28-day Mortality with Area Under the Curve (AUC) from the multivariable model, controlling for age at first encounter, SOFA score, vasopressor use, history of diabetes, history of liver disease, and phosphate quartile. AUC 0.950 (95% CI 0.939, 0.960).

After screening of candidate variables, the LASSO method selected age at first encounter, SOFA score, vasopressor use, history of diabetes, and history of liver disease to be the covariates in the multivariable analyses, in addition to the phosphate quartile. Initial creatinine and calcium levels were included as candidate variables in the initial model. However, because their contribution was non-significant, these variables were not selected by the LASSO procedure for the final model. The area under the ROC curve (AUC) was 0.950 (95% CI 0.939–0.960, Figure 2), indicating excellent performance of the fitted model in predicting 28-day mortality.

**Figure 3:**
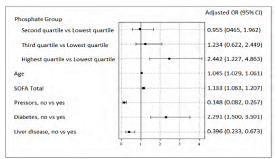
Adjusted odds ratios for 28-day mortality. Odds rations from multivariable model, adjusting for age at first encounter, SOFA score, vasopressor use, history of diabetes, and history of liver disease. *CI*=Confidence interval *OR*, odds ratio; *SOFA*, Sequential Organ Failure Assessment.

Controlling for the effect of these covariates, the repeated-measures mixed-model revealed that the likelihood of 28-day mortality was different across the quartile groups (*P*<0.01). Increasing age, higher initial SOFA score, vasopressor use, and history of liver disease were also significantly associated with mortality (Figure 3).

The odds ratios, adjusted for other covariates in the multivariable model, for 28-day mortality by phosphate quartile groups are presented in Table 3. Accounting for other covariates, the highest phosphate quartile group (> 4.0 mg/dL) had 2.4 times higher odds of death than the lowest (≤ 2.6 mg/dL) quartile group (*P*<0.01), 2.6 times higher odds of death than the second (2.6–3.2 mg/dL) quartile group (*P*<0.01), and 2.0 times higher odds than the third (3.2–4.0 mg/dL) quartile group (*P*=0.04).

#### Sub-analysis of Sepsis-3 Cohort

In the pre-planned sub-analysis of patients with SOFA scores of two or more, initial phosphate remained significantly associated with mortality. There were 1,051 patients in the sub-analysis with an overall mortality rate of 19% (Supplemental Table 1). The mortality rate was highest among patients in the highest phosphate quartile (30%; Supplemental Table 1). Again, patients in the highest quartile had increased odds of mortality compared to those in the lowest two quartiles (Supplemental Table 2). However, mortality differences between patients in the highest quartile and those in the third quartiles were no longer significant (*P*=0.05; Supplemental Table 2). Other significant predictors of mortality in the original model were similar in the secondary analysis multivariable model (Supplemental Figure 1).

## DISCUSSION

In this retrospective study of hospitalized sepsis patients, we found patients in the highest quartile of first 24-hour phosphate levels had increased mortality and odds of death compared to all other phosphate quartiles. Patients in the lowest quartile of phosphate levels had similar odds of mortality compared to those in the second or third quartiles and significantly lower odds of mortality compared to those in the highest quartile. Although we divided phosphate levels into quartiles for analysis, the bounds closely aligned with accepted diagnostic thresholds. The significance of our findings persisted after adjusting for comorbidities, severity of illness, and potential confounders.

Our findings are similar to other recent studies, which have found that elevated phosphate levels are associated with poor outcomes.^16^,^17^ Miller et al reported significantly higher rates of 28-day mortality in mechanically ventilated sepsis patients with phosphate levels above 3.5 mg/dL.^16^ Paradoxically, both time-weighted hypo- and hyperphosphatemia were associated with decreased length of time on mechanical ventilation.^16^ Mortality rates observed by Miller et al were higher compared to our study (58.5% and 28%, respectively); however, all the patients in their dataset were mechanically ventilated and represented a more critically ill cohort at baseline. In our study, which was not limited to mechanically ventilated patients, patients in the highest phosphate quartile (>4.0 mg/dL) experienced higher mortality and were also more likely to require mechanical ventilation.

Al Harbi et al reported increased intensive care unit (ICU) and hospital mortality (aOR 1.60, 95% CI 1.13–2.28, *P*<0.01 and 1.70, 95% CI 1.21–2.29, *P*<0.01, respectively) among ICU patients with hyperphosphatemia.^17^ In addition to increased mortality, patients with hyperphosphatemia had increased vasopressor use and mechanical ventilation dependence.^17^ Similar to our methodology, phosphate levels in their study were from the first day of presentation, although our study was not limited to ICU patients. Although cutoff values in our study were slightly different, and our cohort included a broader severity of illness, we found comparably higher mortality and increased mechanical ventilation requirements among patients with the highest phosphate levels.

Haider et al investigated the association between hyperphosphatemia and mortality among a broad cohort of 2,390 patients presenting to the ED.^25^ They found hyperphosphatemia was an independent risk factor for mortality (OR 3.29; *P*<0.001). Among the 215 patients with hyperphosphatemia, the mortality rate was 10.7%, compared to 3.2% in the overall cohort. Their study was a cross-sectional study of ED patients regardless of the reason for presentation, whereas our study was specifically focused on phosphate derangements among septic patients.

Although we, and others, found septic patients with hyperphosphatemia had higher odds of mortality, some literature supports an association between hypophosphatemia and adverse outcomes.^11^,^12^,^14^ However, there are several notable limitations to those studies. In a small retrospective study of 55 patients from 2006, Shor et al found that severe hypophosphatemia (<1.0 mg/dL) was an independent risk factor for sepsis mortality, compared to patients without severe hypophosphatemia (>1.0 mg/dL).^14^ However, all patients with a phosphate level >1.0 mg/dL were included in the same group for analyses for their study, and no data were presented on patients with hyperphosphatemia.

Suzuki et al included all ICU patients, not limited to sepsis, in their investigation of phosphate levels among critically ill patients.^11^ After excluding patients with any episode of hyperphosphatemia, patients with at least one episode of hypophosphatemia had higher ICU mortality than those without any episodes of hypophosphatemia (34% vs 22%, respectively; *P*<0.01). However, in their multivariable analysis, hypophosphatemia was *not* independently associated with mortality, and a hyperphosphatemia subgroup (>4.3 mg/dL) had increased ICU mortality, duration of mechanical ventilation, and ICU length of stay. Moreover, correction of hypophosphatemia was not associated with improvement of outcomes. Although they did not find hypophosphatemia to be an independent predictor of mortality, their study seems to suggest a potential bimodal distribution, with phosphate extremes (either low or high) having an association with increased illness severity in critically ill patients.

Wang et al also investigated the relationship between hypophosphatemia and mortality among all ICU patients, although not limited to sepsis.^12^ They separated patients into a normal phosphate group and a hypophosphatemia group based on phosphate levels at time of ICU admission and found hypophosphatemia to be an independent risk factor for ICU 28-day mortality (OR 1.5; *P*=0.01). Patients with hyperphosphatemia were excluded from their analysis. We did not find an association between hypophosphatemia and mortality among our cohort of patients with sepsis. Although some studies only investigated severe hypophosphatemia, in our cohort less than 1% of encounters had phosphate levels <1.0 mg/dL. Several of the above studies only examined the relationship between hypophosphatemia and adverse outcomes and excluded patients with hyperphosphatemia. A strength of our study design was that we included patients with both high and low phosphate levels in our analysis.

Our results support some existing literature that suggests an association between hyperphosphatemia and adverse outcomes in critically ill patients. Our study provides valuable insights into the association between phosphate derangements and adverse outcomes in septic patients, an area with limited, and conflicting, existing evidence. Our findings suggest that phosphate dysregulation, and specifically elevated phosphate levels, is associated with increased sepsis mortality, although the pathophysiologic mechanism could not be elucidated by the retrospective design of our study. Potential etiologies of this relationship could include that phosphate is a marker of organ dysfunction or dysregulated cellar metabolism from sepsis. Whether high serum phosphate is an early indicator of increased mortality, a marker of organ failure severity, a potential therapeutic target, or mediates cellular dysfunction, warrants further investigation.

## LIMITATIONS

It is possible that the relationship between elevated phosphate levels and mortality could reflect residual confounding from an unmeasured covariate. For example, increased phosphate could be secondary to other unexplained organ dysfunction that influences sepsis mortality. However, we accounted for a number of potentially confounding factors in our multivariable model and included both SOFA score and markers of renal dysfunction as candidate variables and found high phosphate levels to be persistently associated with increased sepsis-related mortality. Additionally, we had a limited number of patients with severe hypophosphatemia in our patient population, which may have limited our ability to detect significant associations. Finally, given the retrospective nature of our study, we were unable to ascertain the reason a phosphate level was drawn. Although this could have been part of some clinicians’ usual practice, it may also have biased the sample toward a sicker cohort that had a broader laboratory evaluation.

## CONCLUSION

In this retrospective study of hospitalized sepsis patients, we found that patients with the highest initial phosphate levels in the first 24 hours of presentation had increased odds of death compared to patients in all other phosphate-level groups.

## Supplementary Information









## Figures and Tables

**Table 1:** Categorical variables by phosphate quartile group (unique patients).

Variable	Overall	Highest quartile	Third quartile	Second quartile	Lowest quartile	P-value
Age, years*	60 (50, 71)	64 (53, 74)	60 (49, 73)	59 (49, 70.5)	58 (48, 68)	<0.001
Gender, female	950 (51)	219 (54)	233 (52)	238 (52)	260 (48)	0.39
Race, Black	889 (48)	190 (46)	226 (50)	211 (46)	262 (49)	0.34
Other	92 (5)	14 (4)	18 (4)	25 (5)	34 (6)	
White	874 (47)	204 (50)	207 (46)	220 (48)	243 (45)	
AIDS	61 (3)	11 (3)	18 (4)	14 (3)	18 (3)	0.75
Malignancy†	210 (11)	47 (11)	56 (12)	50 (11)	57 (11)	0.83
CHF	388 (21)	127 (31)	85 (19)	92 (20)	84 (16)	<.001
COPD	629 (34)	149 (36)	145 (32)	184 (40)	151 (28)	<.001
CVD	180 (10)	53 (13)	37 (8)	44 (10)	46 (9)	0.08
Diabetes	637 (34)	144 (35)	165 (37)	158 (35)	170 (32)	0.40
Dementia	104 (6)	31 (8)	35 (8)	17 (4)	21 (4)	0.01
Liver disease	207 (11)	47 (11)	45 (10)	53 (12)	62 (12)	0.84
Myocardial infarct	152 (8)	54 (13)	33 (7)	28 (6)	37 (7)	<.001
Mechanical vent	116 (6)	50 (12)	28 (6)	21 (5)	17 (3)	<.001
Metastatic cancer‡	78 (4)	16 (4)	22 (5)	19 (4)	21 (4)	0.87
Vasopressors	532 (29)	204 (50)	105 (23)	111 (24)	112 (21)	<.001
Calcium, mg/dL*^	9.1 (8.6, 9.5)	9 (8.4, 9.5)	9.1 (8.6, 9.6)	9 (8.6, 9.5)	9.1 (8.6, 9.5)	0.07
Creatinine, mg/dL *^	1.09 (0.78, 1.65)	1.73 (1.12, 2.73)	1.11 (0.77, 1.76)	0.94 (8.6, 9.5)	0.99 (0.74, 1.33)	<.001
Lactate, mmol/L*^	2.0 (1.4, 3.1)	2.3 (1.5, 3.9)	1.7 (1.2, 2.8)	1.9 (1.2, 3.0)	2.0 (1.5, 3.0)	<.001
SOFA Total*^	2 (0, 5)	5 (3, 8)	5 (3, 8)	2 (0, 4)	1 (0, 3)	<.001

† Any malignancy except malignancy skin neoplasm;

‡ metastatic solid tumor; data is reported as count (percentage) and analyzed using chi-square test, unless specified by

* median (1st quartile, 3rd quartile) and Wilcoxon rank-sum test;

^ first available measure at first encounter.

*AIDS*, acquired immunodeficiency syndrome; *CHF*, congestive heart failure; *COPD*, chronic obstructive pulmonary disease; *CVD*, cerebrovascular disease; *mg/dL*, milligram per deciliter; *SOFA*, Sequential Organ Failure Assessment,

**Table 2:** Distribution of phosphate quartile groups and mortality by unique patients.

	Phosphate lab value range*	Median phosphate value* (1st quartile, 3rd quartile)	Mortality rate
Lowest quartile	≤2.6	2.20 (1.90, 2.50)	7% (38/539)
Second quartile	2.6 – 3.2	2.90 (2.80, 3.10)	9% (39/456)
Third quartile	3.2 – 4.0	3.60 (3.40, 3.80)	10% (47/451)
Highest quartile	>4.0	5.00 (4.50, 5.90)	28% (113/409)
Overall	0.40–8.20	3.20 (2.50, 3.90)	13% (327/1,855)

* mg/dL.

*mg/dL*, milligram per deciliter.

**Table 3:** Adjusted odds of mortality by phosphate quartile groups.

Phosphate quartile group	Comparator phosphate quartile group	Adjusted OR (95% CI)*	Adjusted *P* value
Second quartile	Lowest quartile	0.955 (0.465–1.962)	0.9984
Second quartile	Third quartile	0.774 (0.377–1.589)	0.79
Third quartile	Lowest quartile	1.234 (0.622–2.449)	0.86
Highest quartile	Lowest quartile	2.442 (1.227–4.863)	<0.01
Highest quartile	Second quartile	2.558 (1.239–5.291)	<0.01
Highest quartile	Third quartile	1.980 (1.026–3.817)	0.04

* From regression model adjusting for age at first encounter, Sequential Organ Failure Assessment score, vasopressor use, history of diabetes, and history of liver disease.

*OR*, odds ratio.
