# Supplementary material for: Association of Early Serum Phosphate Levels and Mortality in Patients with Sepsis
Source: West J Emerg Med. 2023 Apr 28;24(3):416–23. doi: 10.5811/westjem.58959 (PMC10284527; doi:10.5811/westjem.58959)
Supplement: Supplementary file 1 [file wjem-24-416-s001.docx]

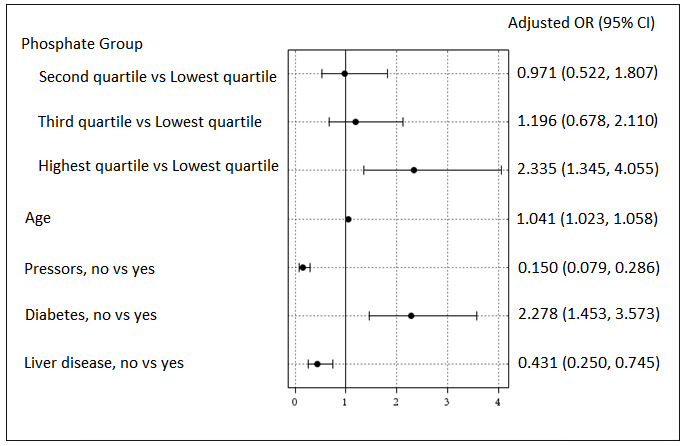


**Supplemental Figure 1: Adjusted Odds Ratios for 28-day Mortality from Sub-Analysis Model**

Odds rations from sub-analysis multivariable model, adjusting for age at first encounter, vasopressor use, history of diabetes, and history of liver disease. OR=Odds ratio; CI=Confidence interval.
