# Supplementary material for: Association of Early Serum Phosphate Levels and Mortality in Patients with Sepsis
Source: West J Emerg Med. 2023 Apr 28;24(3):416–23. doi: 10.5811/westjem.58959 (PMC10284527; doi:10.5811/westjem.58959)
Supplement: Supplementary file 2 [file wjem-24-416-s002.docx]

**Supplement 1: ICD-9 Codes for Sepsis Identification**

785.52 Septic Shock

998.02 Postoperative Shock, Septic

670.2 Puerpural Sepsis, unspecified

670.22 Puerpural Sepsis, delivered, with post-partum complication

670.24 Puerpural Sepsis, postpartum condition or complication

995.91 Sepsis

995.92 Severe Sepsis

659.33 Generalized infection during labor antepartum

659.31 Generalized infection during labor delivered

659.30 Generalized infection during labor unspecified as to the episode of care

054.5 Herpetic septicemia

038.9 Unspecified septicemia

038.8 Other specified septicemias

038.49 Other septicemia due to gram-negative organisms

038.44 Septicemia due to serratia

038.43 Septicemia due to pseudomonas

038.42 Septicemia due to Escherichia coli

038.41 Septicemia due to Hemophilus influenza

038.40 Septicemia due to gram-negative organism unspecified

038.3 Septicemia due to anaerobes

038.2 Pneumococcal septicemia

039.19 Other Staphylococcal septicemia

038.12 Methicillin Resistant Staphyloccocus Aureus septicemia

038.11 Methicillin Susceptible Staphyloccocus Aureus septicemia

038.10 Staphylococcal septicemia unspecified

038.0 Streptococcal septicemia

022.3 Anthrax septicemia

003.1 Salmonella septicemia
