# Supplementary material for: Association of Early Serum Phosphate Levels and Mortality in Patients with Sepsis
Source: West J Emerg Med. 2023 Apr 28;24(3):416–23. doi: 10.5811/westjem.58959 (PMC10284527; doi:10.5811/westjem.58959)
Supplement: Supplementary file 3 [file wjem-24-416-s003.docx]

Supplemental Table 1**:** Sub-Analysis Phosphate Quartiles and Crude Mortality Rates

|  | Phosphate Lab Value Range* | Median Phosphate Value*  (1^st^ quartile, 3^rd^ quartile) | Mortality Rate |
| --- | --- | --- | --- |
| Lowest Quartile | ≤ 2.6 | 2.20 (1.80, 2.40) | 12% (31/257) |
| Second Quartile | 2.6 - 3.2 | 2.90 (2.80, 3.10) | 14% (27/194) |
| Third Quartile | 3.2 - 4.0 | 3.70 (3.40, 3.80) | 16% (40/252) |
| Highest Quartile | > 4.0 | 5.10 (4.60, 6.00) | 30% (106/248) |
| Overall | 0.70-8.20 | 3.50 (2.70, 4.60) | 19% (204/1051) |

*mg/dL
