# Supplementary material for: Association of Early Serum Phosphate Levels and Mortality in Patients with Sepsis
Source: West J Emerg Med. 2023 Apr 28;24(3):416–23. doi: 10.5811/westjem.58959 (PMC10284527; doi:10.5811/westjem.58959)
Supplement: Supplementary file 4 [file wjem-24-416-s004.docx]

Supplemental Table 2**:** Sub-Analysis Phosphate Group Comparisons (from regression model)

| Phosphate Quartile  Group | Comparator Phosphate  Quartile Group | Adjusted OR (95%CI)* | Adjusted P Value |
| --- | --- | --- | --- |
| Second Quartile | Lowest Quartile | 0.971 (0.430, 2.199) | 0.9997 |
| Second Quartile | Third Quartile | 0.812 (0.363, 1.816) | 0.9061 |
| Third Quartile | Lowest Quartile | 1.196 (0.567, 2.525) | 0.9234 |
| Highest Quartile | Lowest Quartile | 2.335 (1.129, 4.828) | 0.0152 |
| Highest Quartile | Second Quartile | 2.404 (1.105, 5.236) | 0.0205 |
| Highest Quartile | Third Quartile | 1.953 (0.998, 3.817) | 0.0508 |
| Note: OR=Odds ratio; CI=confidence interval; *from regression model adjusting for age at first encounter, SOFA score, vasopressor use, history of diabetes, and history of liver disease | | | |
